# Clinical experience with once-monthly risperidone ISM in a mental health center. A retrospective study.

**DOI:** 10.1192/j.eurpsy.2024.1559

**Published:** 2024-08-27

**Authors:** A. Merino Iglesias, N. Rodríguez Ortega, L. Montero Quer, M. R. Rodríguez Campos

**Affiliations:** Psychiatry, Hospital Universitario de Fuenlabrada, Madrid, Spain

## Abstract

**Introduction:**

Long-acting injectable antipsychotics have undergone a great development in recent years, becoming useful tools to facilitate therapeutic adherence. Once-monthly risperidone ISM is a new way of treatment which has been commercialized in Spain since September 2022.

**Objectives:**

In this study we will analyze our clinical experience with this treatment, especially in terms of tolerance and efficacy, during its first year of widespread use.

**Methods:**

Longitudinal retrospective study of monhtly risperidone users in a mental health center in the Autonomous Community of Madrid (Spain), between September 2022 and September 2023.

A sample of 13 patients was selected, collecting both sociodemographic (age, gender) and clinical variables (diagnosis, dose, time elapsed, number of hospital readmissions, adverse effects and monotherapy or combined use). A descriptive analysis of the collected data was then carried out.

**Results:**

Monthly risperidone was used in 13 patients: 15% (n=2) were women, and 85% (n=11) were male. The mean age of the patients was 43.6 years. The most frequent diganosis of these patients was “psychotic disorders” (84,6%, n= 11), with other diagnoses such as schizoaffective disorder (7,7%, n=1) and obsessive compulsive disorder (7,7%, n=1).

The doses used of risperidone were 100mg every month in 61,5% of patients (n=8) and 75mg in 38,5% of patients (n=5). The mean time since the first administration was 4.35 months.

Concerning monotherapy, 84,6% (n=11) of patients on monthly risperidone were on antipsychotic monotherapy, while 15,4% (n=2) required more than one antipsychotic. Among the switches made to monthly risperidone, 69,2% (n=9) were previously treated with oral risperidone, 15,4% (n=2) were treated with once-biweekly risperidone long-acting injectable, 7,9% (n=1) with oral paliperidone and 7,9% with aripiprazole monthly injectable.

During the study period, hospital readmissions for psychiatric descompensations occurred in one patient (7,9%, n=1), while the rest of the patients (92,1%, n=12) did not present decompensations that required psychiatric admission.

Moderate or severe effects occurred in one patient (7,9%, n=1), in the form of acute dystonia, which led to the interruption of injectable treatment. The rest of the patients (92,1%, n=12)) did not present severe adverse effects. Minor adverse effects appeared in 3 patients (25%); these adverse effects were already present in the previous treatment with oral risperidone and did not condition the suspension of the treatment.

**Conclusions:**

In the sample analyzed, once-monthly Risperidone ISM had reasonable tolerance levels. Also, it´s shown to be effective in preventing psychotic decompensations and hospital admissions.

Therefore, this new injectable of monthly risperidone represents a therapeutic alternative to consider in order to guarantee therapeutic adherence and improve the quality of life of patients with psychotic symptoms.

**Disclosure of Interest:**

None Declared

